# Hybridization interactions between probesets in short oligo microarrays lead to spurious correlations

**DOI:** 10.1186/1471-2105-7-276

**Published:** 2006-06-02

**Authors:** Michał J Okoniewski, Crispin J Miller

**Affiliations:** 1 Paterson Institute For Cancer Research, Christie Hospital site, University of Manchester, Wilmslow Road, Manchester, M20 4BX, UK

## Abstract

**Background:**

Microarrays measure the binding of nucleotide sequences to a set of sequence specific probes. This information is combined with annotation specifying the relationship between probes and targets and used to make inferences about transcript- and, ultimately, gene expression. In some situations, a probe is capable of hybridizing to more than one transcript, in others, multiple probes can target a single sequence. These 'multiply targeted' probes can result in non-independence between measured expression levels.

**Results:**

An analysis of these relationships for Affymetrix arrays considered both the extent and influence of exact matches between probe and transcript sequences. For the popular HGU133A array, approximately half of the probesets were found to interact in this way. Both real and simulated expression datasets were used to examine how these effects influenced the expression signal. It was found not only to lead to increased signal strength for the affected probesets, but the major effect is to significantly increase their correlation, even in situations when only a single probe from a probeset was involved. By building a network of probe-probeset-transcript relationships, it is possible to identify families of interacting probesets. More than 10% of the families contain members annotated to different genes or even different Unigene clusters. Within a family, a mixture of genuine biological and artefactual correlations can occur.

**Conclusion:**

Multiple targeting is not only prevalent, but also significant. The ability of probesets to hybridize to more than one gene product can lead to false positives when analysing gene expression. Comprehensive annotation describing multiple targeting is required when interpreting array data.

## Background

Sources of noise in microarray experiments may be numerous [1,2], thus most researchers try to minimize its influence or estimate it through various quality control, normalization and outlier filtering procedures [3]. One source of variation is cross-hybridization (CH), which occurs when unintended sequences hybridize to a probe alongside the intended target. In the case of Affymetrix arrays, which use a set of short (typically 25-mer) oligonucleotide probes to target a transcript, hybridization conditions are carefully controlled with the aim of minimizing the effect of CH due to non-specific binding [4]. In addition, each Perfect Match (PM) probe is accompanied by a Mismatch probe (MM), in which the middle residue has been changed. The intention is that this can be used to provide a measure of the level of CH associated with each PM probe. A more detailed discussion of CH in short oligo arrays may be found in [5]. From October 2004, Affymetrix also started to display brief summaries of cross-hybridization within their own NetAffx service [6].

In some circumstances, probes may match exactly to more than one transcript. This is important because these probes can no longer be identified with a unique transcript, but are instead dependent on more than one gene product. The situation is rendered somewhat more complex by the fact that Affymetrix arrays use more than one probe (typically, 11 PM/MM pairs – together referred to as a "probeset") to target each transcript. Recently, several databases have been built to provide a mapping of Affymetrix probesets to known transcripts [7-10], to sequences from cDNA microarrays [11,12], or for applying algorithmic approaches to cross-platform or cross-species comparisons [7]. A recent paper [13] presents a global overview of the interpretation of GeneChip arrays, and the need to update annotation to match the continued evolution of genomic databases. The solution includes the redefinition of CDF files, similar to what was proposed initially in [10], which may be sufficient in many cases.

The issue of 'multiply targeted' probes is important because they have the potential to result in cross-talk between the probesets they are part of. If their effects are significant, and expression summarizing algorithms are unable to control for them, then one outcome of this will be that otherwise unrelated probesets will appear correlated, since they are being driven by a shared signal.

The ADAPT database [4] was used to investigate the extent and significance of multiply-targeted probesets in Affymetrix expression data (see methods). Use is made of the fact that the platform's combination of short oligos and strict hybridization conditions, which are designed to maximize binding to the PM probes whilst minimizing binding to the MM ones. This makes it viable to use *in silico *methods to identify which probes are likely to bind with 100% identity to which transcripts. We refer to cases of exact matches between probe and transcript as Multiple Targeting (MT), to distinguish from the more general case of cross-hybridization, in which matches with less than 100% identity may occur.

Particular attention is directed at the influence MT can have on the apparent correlation between probesets' expression measurements. Since Pearson correlation is scale independent, it is not influenced by the overall magnitude of either signal being compared, but rather on the similarity in their shapes. Although it may seem counter-intuitive, when two signals are superimposed, the amount of correlation found between each of the original signals and the combined one is driven by the relative variance of those two signals, not by their mean intensity (an example and further discussion of this can be found in the supplemental material). Many microarray data analysis techniques rely on correlation analysis, with the majority of methodologies aiming to draw a distinction between genes that are, in some way, co-occurring, co-expressed or correlated and those that do not follow a significant common pattern. Methodologies such as hierarchical clustering [14,15] and relevance networks [16-18] make direct use of the Pearson correlation coefficient of expression values between probesets, whilst others (such as ANOVA and more general linear models), are ultimately based on correlation-like principles.

## Results

**Figure 1:**
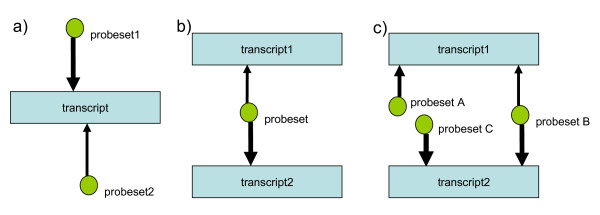
**MT motifs**. The basic motifs of multiple targeting. a) PTP motif b) TPT motif c) a simple combination of both – PTPTP motif. The motifs form the basic building blocks of multiple targeting networks. The strength of relationship between a transcript and a probeset is dependent on the number of probes matching to the transcript.

**Figure 2:**
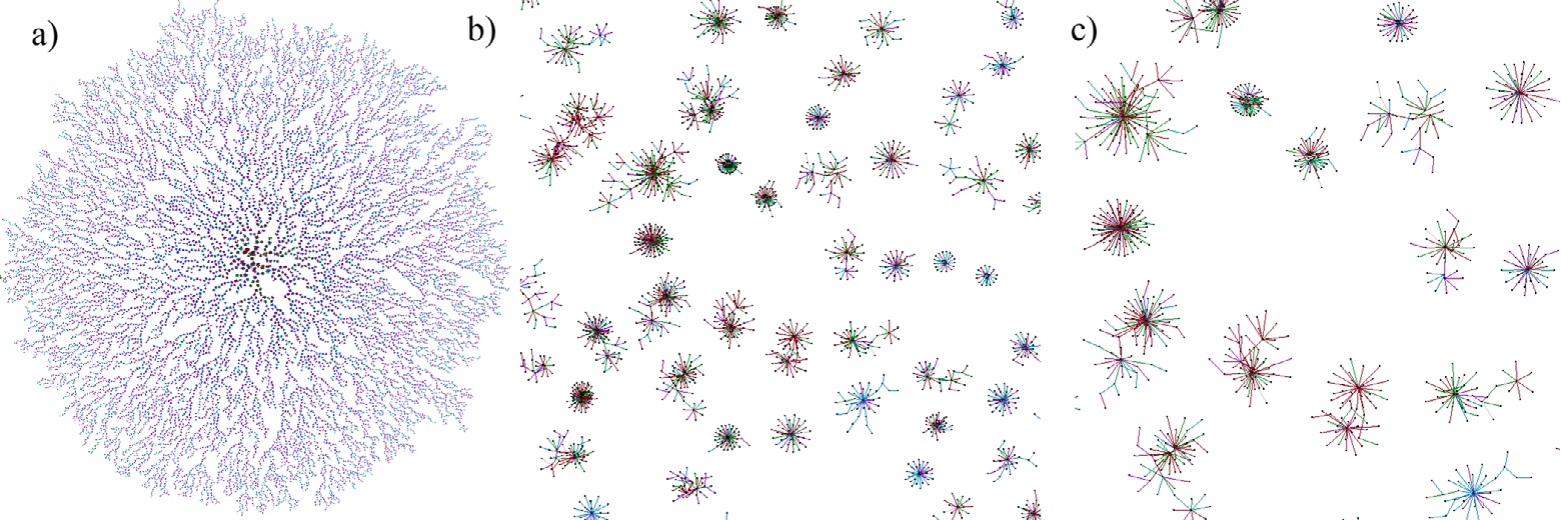
**LGL graph of MT**. a) LGL graph of all probeset-transcript relationships in HG_U133A array b) and c) are close-up views of regions in a)

Affymetrix arrays use a series of probes to target a transcript. These probes are grouped together to form a probeset; expression processing algorithms such as MAS5 [19], RMA [20] and GCRMA [21] combine the signals from each probe in a probeset to provide a single summary value representing an estimate of the concentration of that transcript in solution. The issue of MT arises because certain probes are capable of hybridizing to more than one transcript, leading to non-independence, while in other situations probes from more than one probeset are capable of hybridizing to a single transcript (Figure 1). In general, these interactions combine to form a complex lattice (Figure 2, see also Additional file 1).

In this paper, we consider the extent and structure of these relationships, followed by an investigation of how much effect they have both on signal strength and on the correlation between probesets.

### The prevalence of multiple targeting in oligo arrays

An analysis of the HG_U133A array reveals that many transcripts (Ensembl: 7,257; RefSeq: 6,702) are matched with multiple probesets (i.e. case a in Fig. 1) while almost half (10,223) of the total 22,215 probesets (excluding control probesets) show exact matches (with 1 or more PM probes) to more than one Ensembl (9,460) or RefSeq (9,666) transcript (i.e. case b in Fig. 1). For comparison, 18,722 probesets were found to match to at least one well-known transcript.

The effect of MM probes is minimal: the number of MM probes that can hybridize exactly to known transcripts is about 1,000 times smaller (Ensembl: 1,899 MM matches vs. over 1,956,000 PM matches, RefSeq: 1,962 MM vs. 1,922,000 PM) – most of them singleton matches to unrelated sequences. Thus we exclude MM probes from subsequent analyses. Since MM probes were not considered, and RMA makes no use of these probes in its computations, RMA processed data is used for all calculations presented here, although similar effects were also observed with MAS5 processing.

Affymetrix probeset names are supposed to identify probesets that are associated with multiple targeting. In particular, those marked "_x_at" are identified as being non-specific. Similarly, "_s_at" probesets are identified as potentially targeting different gene family members or splice variants. The analysis shows that many of the probesets associated with MT are not identified in this way and are simply annotated " _at" (2,189 according to Ensembl matches; 1,496 for RefSeq). These numbers are likely to be underestimates because ADAPT was built using only well characterized sequences. Thus, a significant number of the standard "_at" probesets are involved in MT.

### Structures of multiple targeting in oligo arrays

#### Basic motifs

The two basic building blocks of MT interaction networks are Probeset-Transcript-Probeset (PTP) motifs (Figure 1a), and Transcript-Probeset-Transcript (TPT) motifs (Figure 1b). Depending on the robustness of the analysis algorithms used to process array data, the presence of either motif can be expected to lead to non-independence between the expression profiles of the participating probesets.

A search for both types of motifs confirms the prevalence of MT in oligo arrays. Table 1 summarizes the rates of occurrence of both motifs for a variety of Affymetrix arrays. The PTP motif is especially common – it involves almost half the probesets on the HG_U133A array and over a third of those on the HG_U133Plus2. Generally, the more recent arrays have a larger proportion of probesets involved in MT.

#### Families of related probesets

Probesets may be involved in multiple PTP and TPT motifs, resulting in an MT-network. This can be expressed as a graph in which nodes represent transcripts and probesets, while edges represent matches between transcripts and probesets, labelled with the number of matching probes involved in the interaction. Such graphs are informative because so many probesets have the potential to be involved in MT (almost half for HGU133A arrays). Since Affymetrix arrays measure the binding of cRNA sequences to sequence-specific probes, the searches used to define MT help catalogue which binding events are possible. Knowledge of MT interactions is important because it begins to describe what is actually being measured in a microarray experiment.

Figure 2 shows one such graph, laid out using LGL [22]. Edges attached to RefSeq transcripts are painted red, Ensembl ones, green. Blue is used to mark the strength of MT, with intensity corresponding to the number of matching probes. The LGL graph, when magnified, shows a set of disconnected families -detached sub-graphs of various complexity. Thus, almost all of the MT relationships are local ones.

To build families, the database was queried to identify all PTP motifs. Then, a simple search algorithm used to identify the maximal graph that can be reached from a starting probeset using the identified motifs. Probesets that are not involved in any PTP motifs result in trivial families that consist of just a single probeset. An additional step is used to eliminate "hub probesets", as described below.

For HG_U133A arrays, this process results in the identification of 3,859 families containing at least 2 probesets (for examples – see Additional file 2). The mean number of probesets in the family is not high -about 2.56. Interestingly, 429 families (involving a total of 1,529 probesets), were found in which family members were annotated to different genes. Importantly, these families were not simply comprised of "_x_at" probesets: 456 were annotated "_at" and 497 – "_s_at".

A full list of MT families is included in the supplementary data (see Additional file 3), along with an applet that allows the exploration of these families, attached to exemplary expression data (see Additional file 4).

#### Hub probesets

There is a group of probesets (not always annotated by Affymetrix as " _x_at") that match a large number of transcripts, usually with a small number of probes. They may be called "hub" probesets, because their expression combines signals from many available transcripts. In the network of probeset-transcript relationships, hub probesets often join together smaller families of probesets, often many at a time. A typical example of a hub probeset is "221992_at" which matches to 44 RefSeq or Ensembl transcripts, with an average 3.18 probes per match, or "210524_x_at" (127 matches, 1.5 probe on average).

Hubs were selected for the family search algorithm described above if the average number of matching probes was less than 3 and the total number of transcripts greater than 30, or if the total number of transcript matches was greater than 70. This resulted in 277 hub probesets being selected, allowing the granularity of families to be kept to a reasonable level (also see Table 2 for hub selection criteria).

### Quantitation of the effect of multiple targeting

Probes found by the database searches to target multiple transcripts, generally have a higher measured signal than those that target unique transcripts. For example, the average measured expression level in the Gene Atlas data is 16% higher for multiply targeted PM probes and over 80% higher when the PM – MM difference for individual PM:MM probe pairs is considered.

These numbers refer to differences in the raw probe intensities, which are subsequently grouped into probesets and processed by an expression summary tool such as MAS5 or RMA. The following sections investigate whether these changes at the probe level are carried through to the MAS5 or RMA processed expression summaries, and the influence they have on Pearson correlation.

#### Real data, same transcript

**Figure 3:**
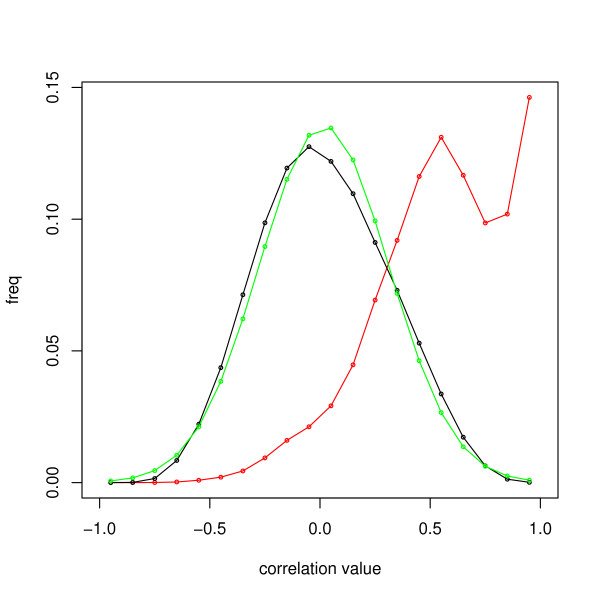
**Influence of MT on correlation between probesets**. The distribution of Pearson correlation for all probeset pairs (black) vs. MT probeset pairs (red). Data from 50 arrays from Gene Atlas processed with RMA. The global (black) curve represents correlation for 1 million random probeset pairs, while the MT curve (red) was drawn using all probeset pairs from over 110,000 PTP motifs that occur in the HG_U133A array. The peak of the MT curve close to a correlation of 1 may be explained by a group of probesets having almost constantly high signal. Most of these are 'hub' probesets as defined in the text. The green distribution is a normal distribution with the same mean ( = 0.018) and standard deviation as the black one. It can be seen that the global distribution is very close to normal.

**Figure 4:**
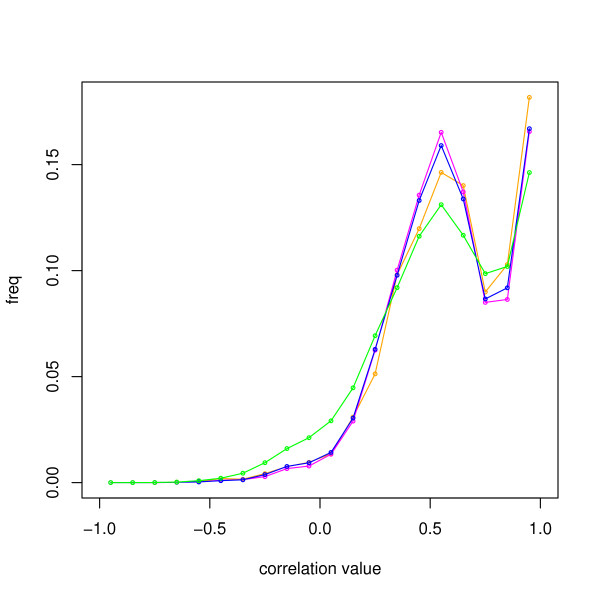
**Effect of MT in real data on correlation between probesets**. Distribution of Pearson correlation for MT-associated probesets. Curves correspond to the number of interacting probes in the PTP motif: orange – 1 probe, magenta – up to 3 probes, blue – up to 7 probes, green – all MT probeset pairs. The peak at the correlation close to 1 is due to hub probesets that generally have high intensity and match to many transcripts with single probes.

Figure 1 draws a distinction between transcripts that share a probeset, and probesets that share a transcript. The first case (PTP, la) is relatively trivial: we should expect to see correlation between these probesets. The extent of the excessive correlation is confirmed by Figure 3, which shows the distribution of the Pearson correlation coefficient calculated between every probeset pair on the array. The resultant distribution is almost normal, with a slight displacement (r¯
 MathType@MTEF@5@5@+=feaafiart1ev1aaatCvAUfKttLearuWrP9MDH5MBPbIqV92AaeXatLxBI9gBaebbnrfifHhDYfgasaacH8akY=wiFfYdH8Gipec8Eeeu0xXdbba9frFj0=OqFfea0dXdd9vqai=hGuQ8kuc9pgc9s8qqaq=dirpe0xb9q8qiLsFr0=vr0=vr0dc8meaabaqaciaacaGaaeqabaqabeGadaaakeaacuWGYbGCgaqeaaaa@2E31@ = 0.02, for Gene Atlas data processed by RMA, for other datasets the mean is comparably small). By contrast, when only multiply targeted probesets are considered (as in Figure 1a), the distribution is strongly distorted towards positive values (r¯
 MathType@MTEF@5@5@+=feaafiart1ev1aaatCvAUfKttLearuWrP9MDH5MBPbIqV92AaeXatLxBI9gBaebbnrfifHhDYfgasaacH8akY=wiFfYdH8Gipec8Eeeu0xXdbba9frFj0=OqFfea0dXdd9vqai=hGuQ8kuc9pgc9s8qqaq=dirpe0xb9q8qiLsFr0=vr0=vr0dc8meaabaqaciaacaGaaeqabaqabeGadaaakeaacuWGYbGCgaqeaaaa@2E31@ = 0.55). Thus, as expected, probesets targeting the same transcript show much higher correlation than those that are not linked in this way. Similar results were also seen with MAS5 and GCRMA processed data (not shown). Importantly, this effect is not confined to probesets in which 11/11 probes match. Figure 4 shows the distribution of Pearson correlation for probesets in which only a subset of probes are involved in MT. It can be seen that even a single matching probe can result in increased correlation. This is surprising given that oligo array data processing methods such as MAS5 and RMA are designed to be robust against outliers – a single probe behaving differently from its peers may not be expected to have a large influence on the data. This is investigated in more detail below.

#### Simulation data

##### Intensity

Figure 1b shows a situation where the expression level of a probeset might be expected to be driven by two different transcripts. Since there is no independent estimate available for the expression levels of the individual transcripts involved in TPT motifs, simulation experiments were performed to mimic the effect by artificially spiking raw expression data.

**Figure 5:**
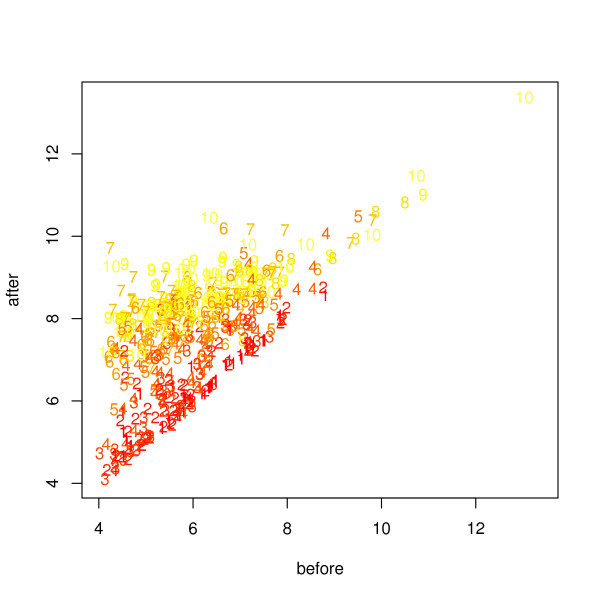
**Simulation experiment – fold change**. Change in measured signal intensity following spiking to simulate the presence of an additional hybridizing transcript in equal abundance to the intended target. Numbers denote the quantity of probes modified in a probeset. Axes are *log*_2_. Even a single spiked probe can result in significant change of the intensity. Fold changes can be seen even for high intensity target probesets.

Figure 5 shows the results of one such simulation, designed to consider the effects of the presence of an additional transcript in equal abundance to the intended target. It can be seen that as the number of spiked probes increases, the signal becomes more pronounced. As previously observed with real data, a single matching probe can have a significant influence on the computed expression level. Even when the expression level is relatively high the signal from only 2 probes can be sufficient to lead to apparent differential expression. Even so, the largest fold changes are generally restricted to the lower intensity probesets, indicating that both MAS5 and RMA do a good job at reducing the effects of outliers.

##### Correlation

**Figure 6:**
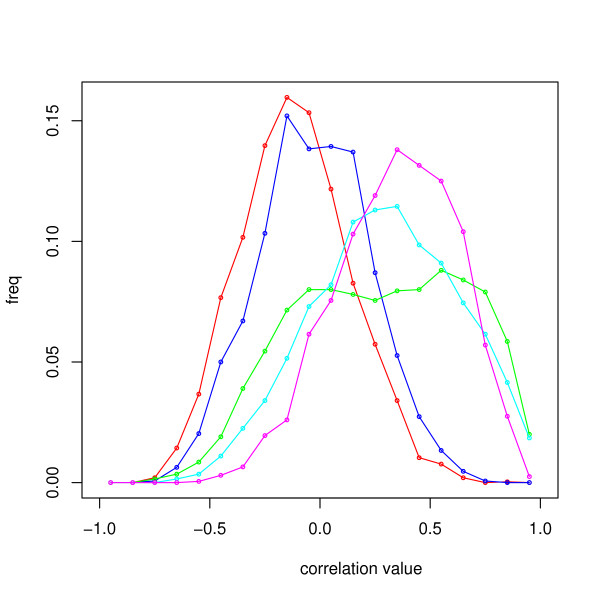
**Variance filtering of spikes and target probesets**. The distribution of correlation for data generated as in Figure 5, but grouped according to variance. Green – high variance probeset plus high variance spiking, blue – high variance probeset plus low variance spiking, magenta – low variance probeset plus low variance spiking, cyan – low variance probeset plus high variance spiking. Red – correlation before spiking. The effects of multiple targeting on correlation is most pronounced when the intended target is of low variance, however even in the case of high variance targets, correlation is likely to be influenced.

In a second simulation experiment, spiking was achieved by adding the signals from a second set of probes to the first set. In this way, the case shown in Figure 1b was simulated – i.e., a probeset hybridizing to two different transcripts (one with all the probes matching, the other with a varying number of matches). The second group of probesets was produced by randomly selecting up to 500 probesets. Variance filtering was performed to ensure that at least one of the transcripts had an expression profile that varied. Since Pearson correlation is not dependent on the mean intensity of the signals, but rather the similarity of their shapes, filtering was performed on variance, not intensity. Pearson correlation, *r *was calculated between each member of the first list and its corresponding partner in the second. Before spiking, the two sets should be uncorrelated; spiking is expected to increase correlation. As in the real data, the signal from the spiked probes contributes significantly to the correlation, even when only a small number of probes are involved. It can be seen from Figure 6 that even when high variance probesets are the recipients of additional spiked signals changes in *r *are possible. Thus, the effects are not restricted to probesets with low signal (see also Additional files 5, 6, 7, 8).

#### Intensity vs. correlation

Both real and artificial datasets demonstrate that MT can have a significant effect on correlation, even when only a small proportion of probesets are involved in the interaction. Algorithms such as RMA and MAS5 successfully employ robust averaging techniques (such as median polishing or a Tukey's biweight) to reduce the effect of outliers. Thus, when only a small number of probes in a probeset are involved in MT, changes in measured expression level are expected to be generally small. This is confirmed in both the real and simulation datasets.

However, even when overall changes in intensity are minimal, increase in Pearson correlation can still be high. This is because Pearson correlation is driven by similarity in profile shape, not intensity; small amounts of stray signal can lead to large increases in *r*, even if the overall mean between probesets are very different. Since Pearson correlation centers each variable about its mean, and scales it by its standard deviation, correlation is entirely dependent on the relative shape and variance of the two signals, not their overall intensity. When two signals, *a *and *b *are compared to their sum, *s*, the signal that is most correlated with *s *depends not on their relative sizes, but on their relative variance. This is counter-intuitive but important to recognize when considering the effects of interacting signals on correlation (see Additional file 9).

**Figure 7:**
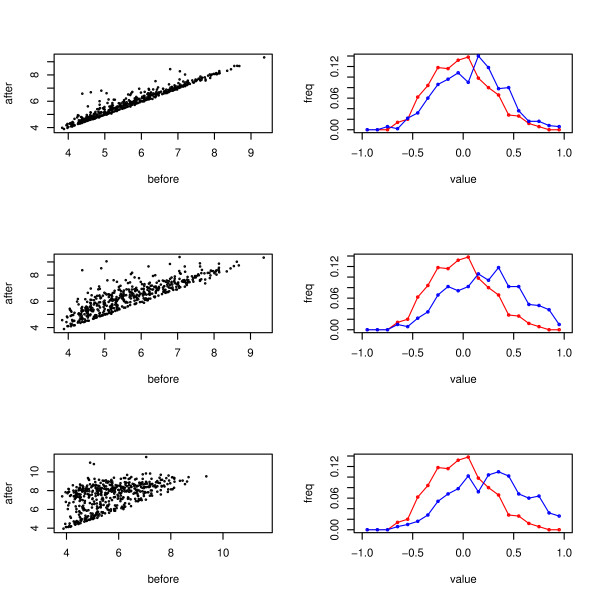
**Influence of the level of spiking on RMA expression values and distributions of correlation**. 1st row: *f f *= 0.05, 2nd row: *f f *= 0.2, 3rd row: *f f *= 1. 1st column scatterplot of the signal after RMA versus the signal before spiking, 2nd column distortion of the correlation distribution – changes after spiking. 500 randomly selected targets and spikes. Even small amount of stray signal may significantly influence correlation. For *f f *= 0.2, the fold change is not so much affected, but the effect on correlation distortion is almost as large as for *f f *= 1.

This effect can be demonstrated by varying the amount of contribution made by the spiking probes (*f f *-see Methods) to the resulting value. Figure 7 shows that even when only 5% of the spike signal was present, the influence on Pearson correlation can still be large, even though the resultant fold change is generally small.

Together Figures 6 and 7 show that increases in correlation are not simply confined to those cases in which a large and varying signal is being added to a low-variance probesets.

In situations where probesets are already strongly correlated, the addition of extra signal due to cross hybridization with another transcript might be expected to reduce correlation. Spiking experiments found this to be the case (data not shown). Interestingly, however, even though there are occasions where *r *is reduced by multiple targeting, the general tendency is towards significantly increased correlation (as shown in Figure 3; similar figure for simulation experiments – see Additional file 10).

False positive rates will also be raised because otherwise absent probesets with signals resulting only from background levels of non-specific hybridization can experience additional, structured, signal due to exact matches to transcripts other than the intended target.

### Functional homogeneity and spurious correlations in families of probesets

Analysis of MT-families shows that out of the 3,859 shown in Figure 2, 395 contained probesets annotated (using the BioConductor annaffy package [3]) to 2 or more UniGene clusters. When gene symbols are considered, annotation becomes even more ambiguous: 429 families contained transcripts annotated to different genes. Thus, even though the majority of families are homogenous with respect to UniGene and gene symbols – some 10–15% (depending on size of the family and source of annotation) may be annotated to different genes. This translates to about 1000 probesets.

**Figure 8:**
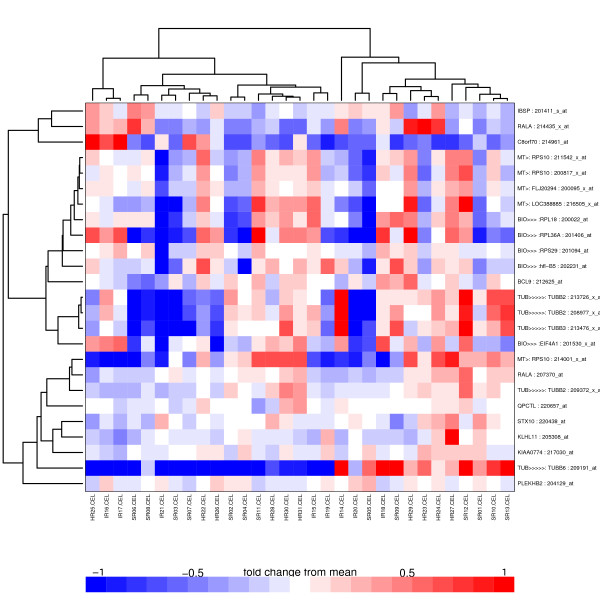
**Heatmap example**. Heatmap and hierarchical clustering of 3 families of probesets (MT-driven, tubulin and a functional one), plus randomly chosen non-MT probesets. The clustering does not make any distinction between functional and MT families – is grouping them together in very similar way.

As we have shown using both real and artificial data, MT leads to increased correlation. A consequence of this is that probesets associated by MT should be drawn closer to one another in dendrograms, such as those used to cluster probesets for visualisation using heatmaps. For example, the heatmap in Figure 8 was created using three groups of probesets. The first (annotated to the genes RPS29,HFL-B5,EIF4A1,RPL36A and RPL18), was identified in [23] as discriminating between standard-risk and high-risk TEL-AML1 cytogenetic abnormalities. Non of these probesets are associated by MT and can thus be considered to form a "well behaving" biological family. The second set (annotated to TUBB6, TUBB2 and TUBB3) constitute another biological family, but they are also associated by MT to each other, as well as to other tubulin genes. This family thus represents a mixed biological – MT family. The third group of probesets are associated with RPS10, but also to numerous pseudogene transcripts. This group represents an "MT family" where the relationship is expected to be artefactual. These three sets of probesets were added to a further set of randomly selected probesets, to act as "background", and then clustered. The MT-family, the tubulins and the biological family are found as separate clusters (the MT-family with even closer links than others), demonstrating that the hierarchical clustering is unable to make a distinction between probable real (i.e. biological) and probably artefactual (i.e. MT driven) clusters.

## Discussion

It is clear that multiple targeting is an important artefact within microarray data: nearly half of all probesets on the HG_U133A array are associated with MT. When real expression data are considered, it can be seen that these probesets are significantly more correlated than would be expected by chance. These results are also supported by simulation experiments, using datasets derived from real experimental data, that allow MT to be considered in a more controlled framework. MT can lead to increased correlation between associated probesets, even when only a small proportion of their probes are involved. Although expression summary algorithms are successful at reducing the effects of outlier probes, the do not remove them completely, and small amounts of stray signal can still have a significant influence on correlation. The reason for this apparent paradox is the scale-invariance of Pearson correlation; absolute signal is not important. What is important are the variance and (effectively) the relative similarity in shape of the expression profiles. For this reason, particular care must be taken when analysing expression data using correlation-based approaches. The situation is also further complicated by the fact that MT occurs at a probe level – adding additional signal to individual probes within a probeset – but correlation is calculated after normalization and expression summarization using an algorithm such as RMA or MAS5. This additional complexity makes it difficult to reliably predict what will happen when signals are combined. However, empirical data (Figure 6) show that influence on correlation is dependent on the relative variance of the two probesets being combined. As expected, high variance spiked probes generally have more of an effect than low variance spikes, but interestingly, adding low variance spikes to low variance data (the magenta line in Figure 6) has more of an effect than adding high variance spikes to low variance data (the cyan line). This is likely to be a consequence of the expression summarization and normalization that is imposed on the data.

One consequence of MT is that because it serves to add structure to otherwise random probesets with no genuine signal, it can lead to the detection of false positives unless the presence of cross-matching probesets is known. Analysis of the intensity distributions for MT and non-MT probesets shows a considerable degree of overlap (see Additional file 11). This means that MT probesets cannot be removed simply by filtering on intensity. In fact, because MT generally increases signal strength, such filtering might actually serve to enrich for MT probes.

MT is ultimately a sequence-based event; it occurs when two sequences show 100% identity across the 25bp targeted by a probe. At the level of a probeset, this is most likely to occur when transcripts show a high degree of sequence similarity. The relationships is troublesome, because a major use of expression data is to identify probesets (and, via annotation, genes) with correlated expression profiles, and to use these relationships to infer functional similarities. Since sequence similarity is itself often the basis by which common function is inferred [24], sequence similarity combined with MT has the potential to become a self-fulfilling prophecy.

A search of the database found that about 5% of family members contained probesets annotated to different genes. Thus, the chances of finding a spurious functional relationship due to MT between a pair of randomly selected genes is small. However, this is optimistic, because microarray analysis generally involves filtering to produce a set of significant probesets (either by magnitude of change, or by statistical confidence). The result of such filtering is to enrich the final 'hit list' not only for real biological effects, but also for anything else that is consistent, including biochemical or sequence based artefacts such as MT. This is illustrated by the heatmap in the Figure 8; MT families fall into separate clusters against a background of randomly selected probesets.

One possible solution to MT is to redefine probesets so that probes targeting the same transcript are placed into larger probesets representing the entire sequence, as proposed by [10]. This is the approach taken also by [13], but authors conclude that "under many circumstances it is not possible to generate transcript-specific probe sets for genes with multiple transcripts based on probes available on the current generation of GeneChips". Thus they may be used to make distinction at the level of genes, but not at the level of transcripts or splice variants – MT with all its consequences, still exists. There is a compromise to be made between generalisation and maintaining the ability to resolve subtle differences between transcripts and, for example, splice variants.

The issue becomes more significant with the new generation of microarrays such as the Affymetrix exon array [25] that deliberately use multiple probesets to distinguish between individual transcripts from within a set of splice variants expressed by a particular gene. The result is a many-many relationship between gene, transcript and probeset.

Annotation schemes that attempt to compress these many-many relationships into a one-to-one mapping lose the complexities inherent in the system. Grouping together a probeset that targets more than one transcript with probesets that target one or more individual transcripts, results in MT occurring between the new probeset and all the other transcripts it shares probes with. From one perspective, many these issues are simply down to annotation. The apparent aretefacts in the data only exist because the probeset annotations do not accurately reflect the transcripts they bind to.

With all solutions, including those that attempt to solve the problem by aggregating probes into larger probesets, annotation is crucial, since inaccuracies will arise unless all the many-many relationships that occur within the data are represented explicitly.

## Conclusion

Cross hybridization between probesets is a significant effect that has real consequences for the interpretation of microarray data. It may cause a variety of problems during analysis including false positives and negatives, and generally increased correlation between multiply-targeted probesets. Although the results presented here are for Affymetrix arrays, it is reasonable to expect similar effects to occur with other expression-based technologies. The use of short oligos and strict hybridization conditions makes it possible to perform the *in silico *searches required to identify MT within Affymetrix data. However, CH is not exclusive to any one platform, and similar behaviour is likely to be seen elsewhere. Expression summary algorithms must correct not only for variation across arrays, but also for variation between individual probes within a probeset. This is generally performed using some kind of robust averaging procedure, but even small amounts of stray signal can lead to high correlations between probesets. Although algorithms such as RMA and MAS5 do a very good job of significantly reducing the influence of outlier probes, they do not always remove it completely – and this is manifested by significantly increased correlation between probesets, even when only a small subset of probes are involved.

Many of the issues described above can be avoided with more detailed annotation. Often the terms 'gene', 'transcript' and 'probeset' are used interchangeably. This is dangerous, because the relationship is not one-one-one, and the existence of MT networks can lead to apparent biological relationships that are, in fact, artefactual. Expression data that is presented simply as a gene list is difficult to interpret properly, since the complexities of the interaction networks implicit within the data are lost. The community should ensure that the actual probeset IDs are always available alongside gene names or transcript accessions. This allows the graph structures associated with gene-transcript-probeset mappings to be explored where necessary and used to fully interpret the complexities of gene expression data.

## Methods

### Graph rendering

MT networks and interaction graphs were produced by extracting data from ADAPT and redirecting the output for visualization to LGL [22], and our own visualization software. As global layouts of graphs such as LGL are static, thus not interactive, because the number of vertices is too big for efficient real-time rendering, an applet was developed for fast and flexible analyses of individual families. These small, local graphs within the applet were realized with the JUNG API [26].

### Databases, experimental data sources and data processing

ADAPT [4] is a database of mappings between Affymetrix probesets, transcripts and genes. It is populated by searching all probe sequences for exact matches to transcript data taken from RefSeq (Release 11 at the time of writing) [27] and Ensembl (V30 at the time of writing) [28]. For RefSeq, both "known" and "model" sequences are used; for Ensembl, ADAPT uses those assigned "known", "novel" or "pseudo" status. Both databases are used because they employ different methods to predict transcript/gene sequences.

The ADAPT database was queried (using SQL and RdbiPqSQL database link to R) to extract a set of tables describing all possible MT links between probesets and transcripts, excluding anti-sense strand matches. The probesets may match transcripts with anything from 1 to 16 matching probes. The tables implicitly define an unconnected graph (see Figure 2 and supplemental file 1), and form the basis for all subsequent explorations of MT. In order to consider the strength of the MT effect and its consequences on expression studies, data from ADAPT were combined with expression data from experiments generated using the HG-U133A array. Expression levels were produced using MAS5 and RMA, as implemented in Bioconductor (packages *affy* and *simpleaffy *[3,29,30]).

Results were analogous when experiments were repeated with MAS5. All plots presented were generated using the Novartis Gene Atlas [31] dataset. Similar results were seen with both leukaemia [23] and sarcoma [32] datasets – publicly available from ArrayExpress.

Pearson correlation was calculated for all the pairs of probesets found to be targeting the same transcript. The distribution of correlation coefficients was calculated for all probeset pairs and for all pairs where one of the probesets matches to a transcript with less than a specified number of probes.

### Simulation data

A subset of 50 HG_U133A arrays from Gene Atlas V2 was used as the basis for simulation experiments designed to explore how the number of MT probes influences expression measurements from RMA processed data.

Spiking was conducted as follows: prior to expression summary generation using RMA, 500 probesets were selected at random to be spiked and 500 (at random) to act as a source of spiking data. No filtering was applied to these probesets. Probesets were randomly paired, and between 1 and 10 probe-pairs selected for each probeset (again at random). The signals from the spike-sources were added to the original signals for the spike-targets. In this way TPT motifs were simulated. The resulting simulated data were batch normalized using RMA and compared to the original un-spiked data (again batch normalized using RMA, separately from the first set). In all simulation experiments spiking for the selected probesets was carried out across the entire set of arrays.

In the second experiment, a set of 500 probesets was selected, as before. A second set with the same number of probesets was then chosen at random. These probesets were selected from a subset of the probesets available, generated by filtering the expression data on variance. In this way, both sets could be sampled from probesets with specifically high, average or low variance of expression. High and low variance are defined as the top or bottom 2000 probesets, sorted by of variance, excluding the 100 most extreme ones. Each probeset in the second list was used to supply data for the probeset in the first list; between 1 and 11 probes were chosen and the probe intensities from the second list added to the corresponding probes in the first list. Various levels of influence were applied, adding a specific proportion of one probeset signal to another: *PM*1_1*after *_= *PM*_1*before *_+ f f * *PM*_2_, where for *f f *ranging from 0.05 to 1. In this way, cross-hybridization between probesets in the first list, and the transcripts represented by the probesets in the second list, was simulated.

## Abbreviations

ADAPT – "A Database of Affymetrix Probesets and Transcripts"

CH – cross-hybridization

LGL – Large Graph Layout

MAS5 – MicroArray Suite – Affymetrix algorithm (MAS 5.0)

MM – mismatch probe

MT – multiple targeting

PM – perfect match probe

PTP – probeset-transcript-probeset network motif

RMA – Robust Multichip Average algorithm

TPT – transcript-probeset-transcript network motif

## Authors' contributions

MO developed the concept of interactions in families of probesets, carried out database and statistical analyses and drafted the manuscript, CM conceived the study on probes alignments to transcript and its implications, supervised and participated in its design and helped to draft the manuscript.

## Supplementary Material

Additional File 1 Graph of MT families for HGU133A array. Animated GIF, 3D visualization of MT-families in the array. Click here for file

Additional File 2 Examples of 3 families of probesets and transcripts. Screenshots from the applet. Big nodes signify probesets (green – positive detection call), small magenta ones – transcripts. The width of edges is proportional to the quantity of MT probes. Probes are marked with a name, annotation in Affymetrix or BioConductor and expression values. Presented are families associated mainly with PAX8, RUNX1/RPL22 and tubulins. Click here for file

Additional File 3 List of MT families for HGU133A array. The CSV file lists all the discovered MT probeset families along with their gene-level annotations according to Affymetrix and BioConductor. Click here for file

Additional File 4 Applet for families exploration. . An applet for browsing graphs of MT-families in the HGU133A array. Big nodes represent HGU133A probesets: green ones have "Present" detection call, pink ones "Absent". They are labelled with Affymetfix and BioConductor annotations, detection call and expression value in the experiment. Small magenta nodes represent transcripts. There is a possibility to add Exon 1.0ST probesets (blue) to the graph. The width of edges is proportional to the number of matching probes. The applet is intended for online use – it is connected to an application server and ADAPT database. Click here for file

Additional File 5 Spiking experiment, signal filtering 1. Scatter plot and correlation distribution, generated as in Figure 7, but filtered by average signal intensity. Low intensity: the 10% probesets with lowest mean signal. High intensity: the 10% probesets with highest mean signal. Low intensity spikes added to high targets. The plots in Additional files 5, 6, 7, 8 prove that with any sort of signal intensity filtering, the shift in correlation coefficient occurs. Click here for file

Additional File 6 Spiking experiment, signal filtering 2. As Additional file 5, but high intensity spikes added to high targets. Click here for file

Additional File 7 Spiking experiment, signal filtering 3. As Additional file 5, but high intensity spikes added to low targets. Click here for file

Additional File 8 Spiking experiment, signal filtering 4. As Additional file 5, but low intensity spikes added to low targets. Click here for file

Additional File 9 R code for correlation experiment. A simple experiment to consider the relationship between correlation cooefficient and variance using 10,000 randomly generated cases. Click here for file

Additional File 10 Influence of specific number of spiked probes on correlation. Changes in Pearson correlation following spiking to simulate MT between probesets. Red plot – correlation before spiking, orange – 1 spiked probe per probeset, magenta – up to 3 probes, blue – up to 7 probes, green – all probes spiked. As in the case of real data – even a single probe may influence the distribution of correlation, however in that case there are no effects of biological similarity – that's why the effect exists, but is smallest for single probes. Click here for file

Additional File 11 Distribution of the expression signal for MT and non-MT probesets after processing with RMA and MAS5. The plots (normalized distributions of summarized expression values) indicate a slight increase in the high signal values for MT probesets (blue) against non-MT probesets (green). Click here for file

## Figures and Tables

**Table 1:** Summarization of PTP and TPT motifs for various Affymetrix arrays

	PTP motifs			TPT motifs		
array	p-set pairs	probesets	% probesets	transcripts	probesets	% probesets

hgu133Plus2	78504	18182	33.29%	11576	7285	13.34%
hgu133a	54762	9666	43.51%	9288	5289	23.81%
hgu133b	6218	3197	14.16%	3241	1526	6.76%
hgu95c	70156	1620	12.88%	3201	928	7.38%
hgu95b	2930	1729	13.77%	2256	1042	8.30%
hgu95e	2984	1515	12.05%	2113	897	7.13%
hgu95d	1444	612	4.87%	1765	502	3.99%
hgu95a	15320	3880	31.32%	7309	3085	24.91%
moe430_2	30552	14234	31.61%	4995	2439	5.42%
moe430a	21686	9808	43.35%	4142	1929	8.53%
moe430b	4224	2647	11.76%	1155	517	2.30%
mgu74av2	17142	2843	22.89%	3221	1184	9.53%
mgu74bv2	3606	2302	18.55%	1175	521	4.20%
mgu74cv2	1158	636	5.36%	888	243	2.05%
Rat230_2	7134	4818	15.52%	2821	1011	3.26%
rae230a	2812	2056	12.96%	2404	792	4.99%
rae230b	1792	1293	8.46%	581	226	1.48%
rgu34b	908	680	7.79%	681	181	2.07%
rgu34c	1382	769	8.81%	745	243	2.78%
rgu34a	14818	3373	38.59%	2302	752	8.60%
rnu34a	1050	600	47.51%	171	81	6.41%
rtu34a	1130	442	45.47%	255	120	12.35%

**Table 2:** Number of hub probesets and hub probesets not annotated x_at depending on the condition of the number of matching transcripts

Transcripts matched – more than:	hubs	non-x_at hubs
10	1548	630
15	1020	289
20	762	144
25	613	96
30	531	73
35	450	56
40	404	47
45	378	42
50	334	29
