# Supplementary figures and images for: Hybridization interactions between probesets in short oligo microarrays lead to spurious correlations
Source: BMC Bioinformatics. 2006 Jun 2;7:276. doi: 10.1186/1471-2105-7-276 (PMC1513401; doi:10.1186/1471-2105-7-276)

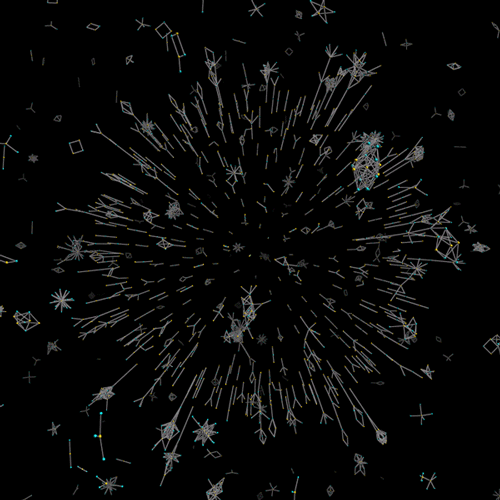

Supplement: Additional File 1 — Graph of MT families for HGU133A array. Animated GIF, 3D visualization of MT-families in the array. [file 1471-2105-7-276-S1.gif]

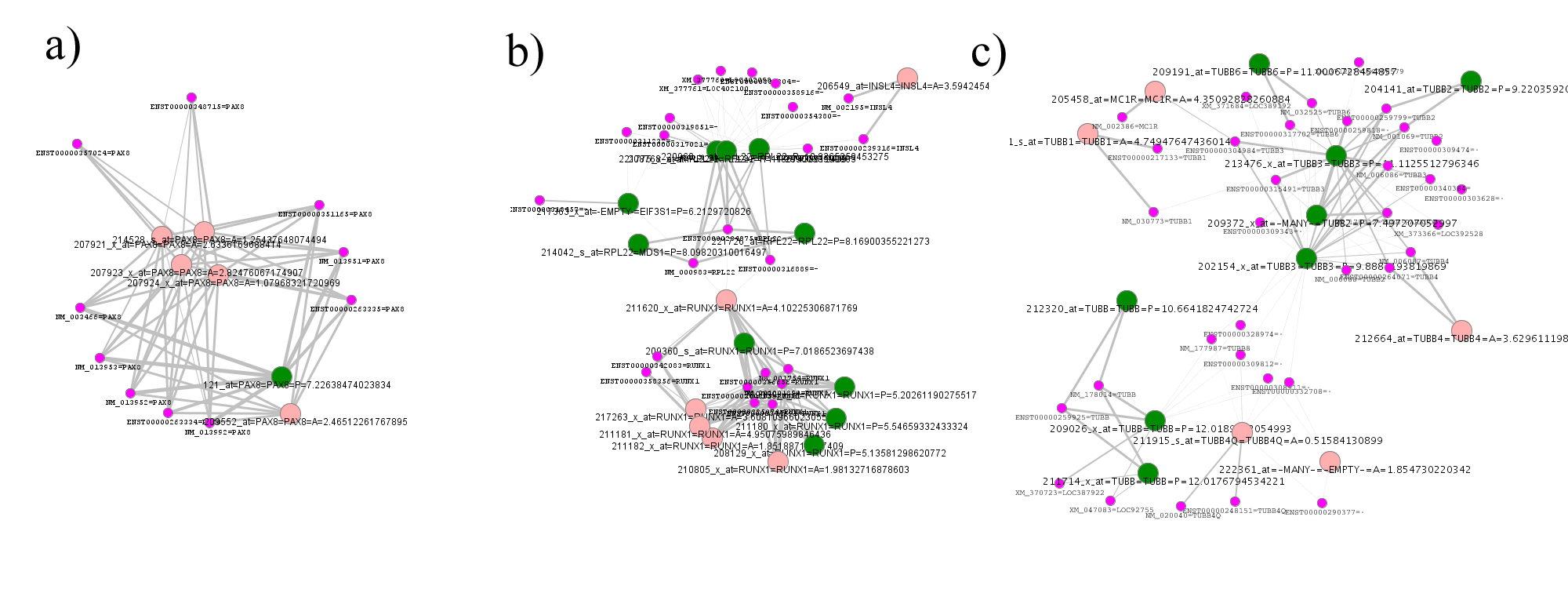

Supplement: Additional File 2 — Examples of 3 families of probesets and transcripts. Screenshots from the applet. Big nodes signify probesets (green – positive detection call), small magenta ones – transcripts. The width of edges is proportional to the quantity of MT probes. Probes are marked with a name, annotation in Affymetrix or BioConductor and expression values. Presented are families associated mainly with PAX8, RUNX1/RPL22 and tubulins. [file 1471-2105-7-276-S2.tiff]

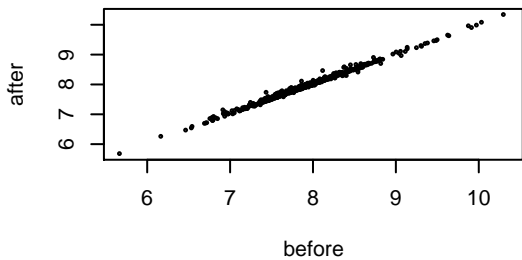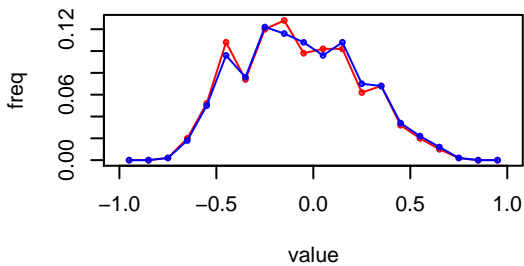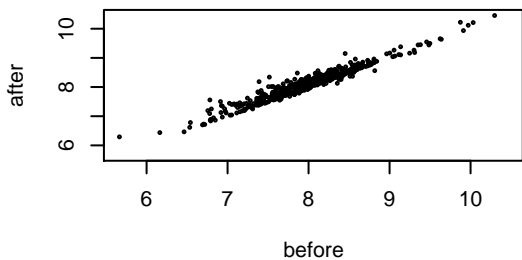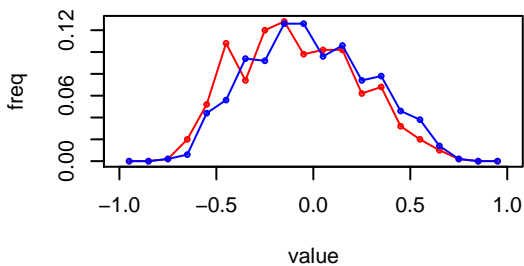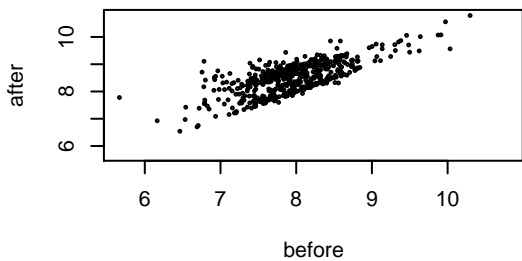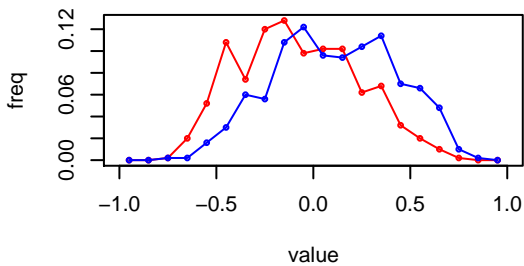

Supplement: Additional File 5 — Spiking experiment, signal filtering 1. Scatter plot and correlation distribution, generated as in Figure 7, but filtered by average signal intensity. Low intensity: the 10% probesets with lowest mean signal. High intensity: the 10% probesets with highest mean signal. Low intensity spikes added to high targets. The plots in Additional files 5, 6, 7, 8 prove that with any sort of signal intensity filtering, the shift in correlation coefficient occurs. [file 1471-2105-7-276-S5.pdf]

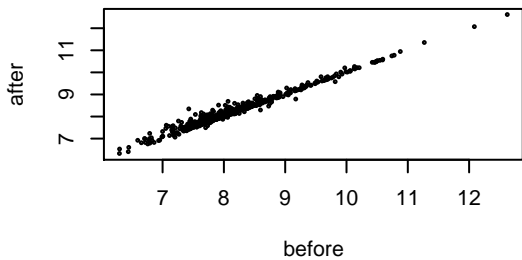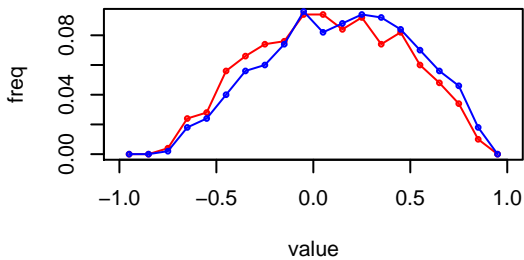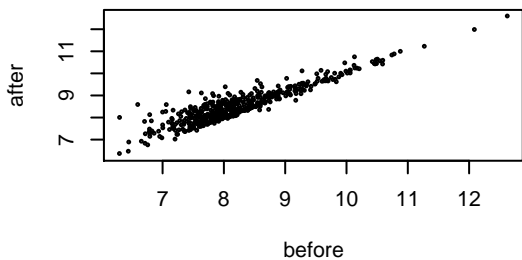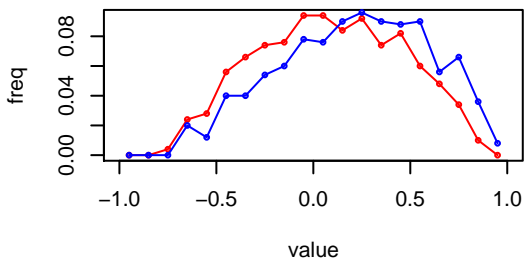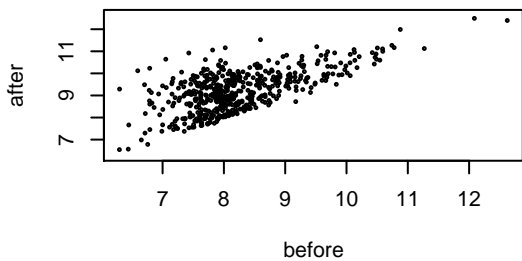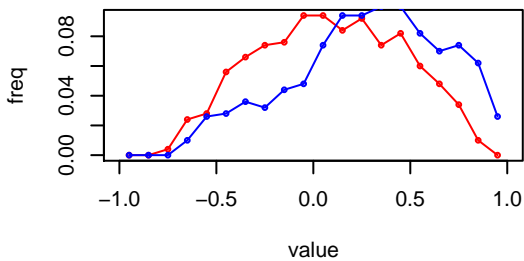

Supplement: Additional File 6 — Spiking experiment, signal filtering 2. As Additional file 5, but high intensity spikes added to high targets. [file 1471-2105-7-276-S6.pdf]

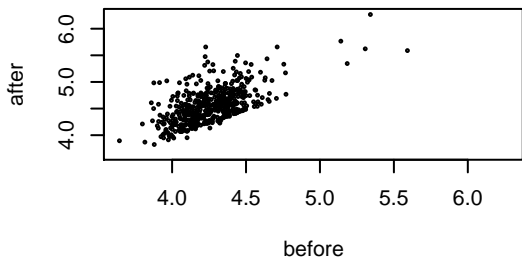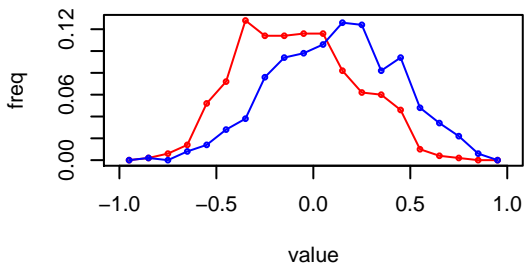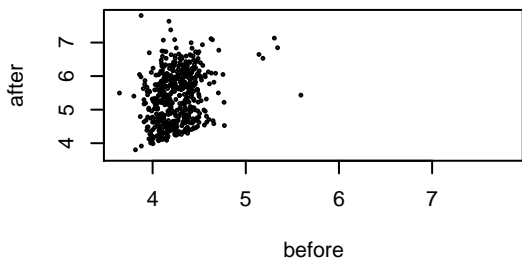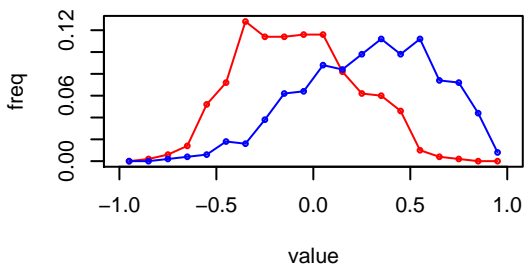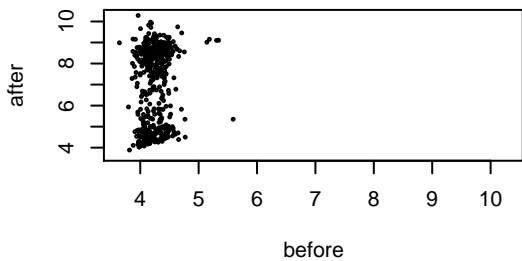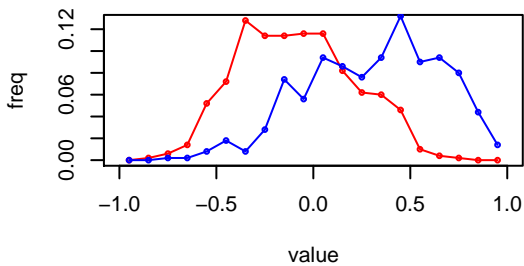

Supplement: Additional File 7 — Spiking experiment, signal filtering 3. As Additional file 5, but high intensity spikes added to low targets. [file 1471-2105-7-276-S7.pdf]

after

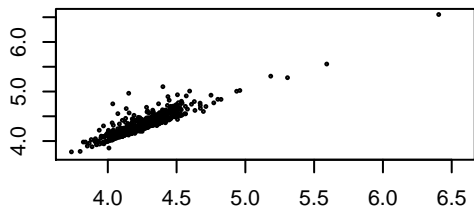

before

freq

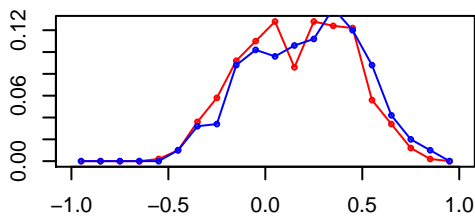

value

after

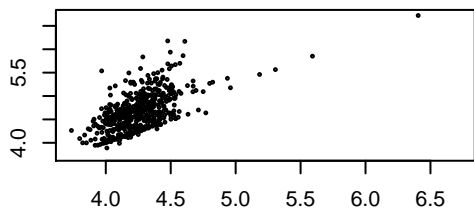

before

freq

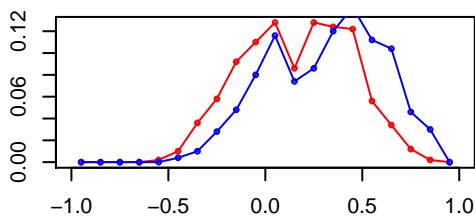

value

after

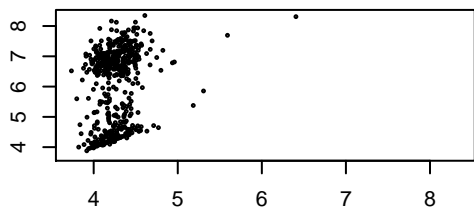

before

freq

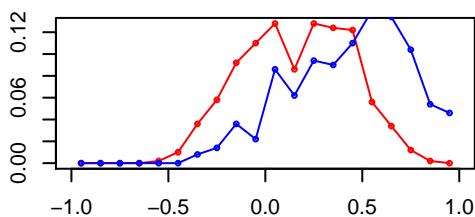

value

Supplement: Additional File 8 — Spiking experiment, signal filtering 4. As Additional file 5, but low intensity spikes added to low targets. [file 1471-2105-7-276-S8.pdf]

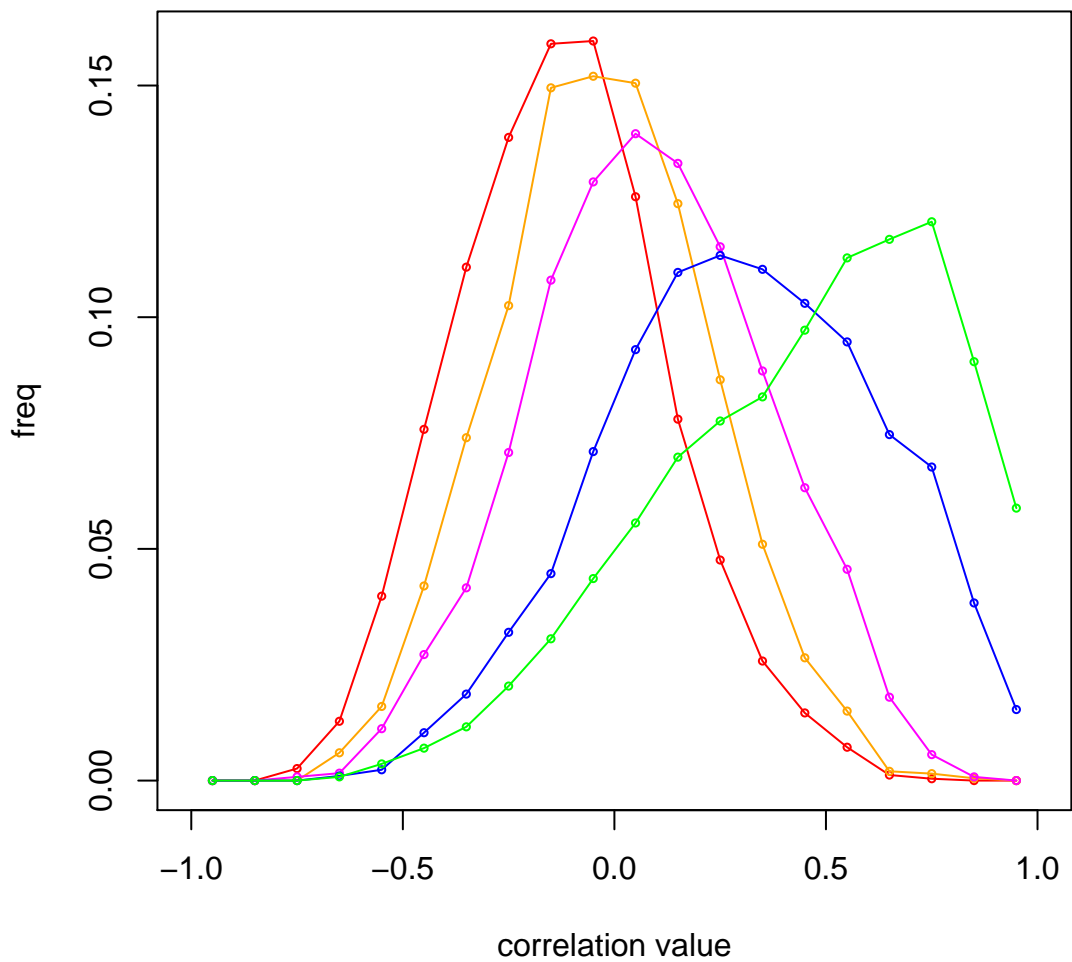

Supplement: Additional File 10 — Influence of specific number of spiked probes on correlation. Changes in Pearson correlation following spiking to simulate MT between probesets. Red plot – correlation before spiking, orange – 1 spiked probe per probeset, magenta – up to 3 probes, blue – up to 7 probes, green – all probes spiked. As in the case of real data – even a single probe may influence the distribution of correlation, however in that case there are no effects of biological similarity – that's why the effect exists, but is smallest for single probes. [file 1471-2105-7-276-S10.pdf]

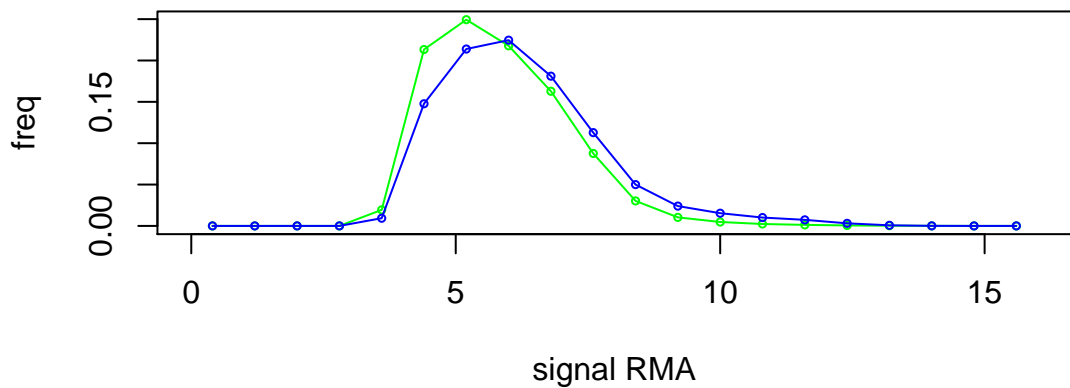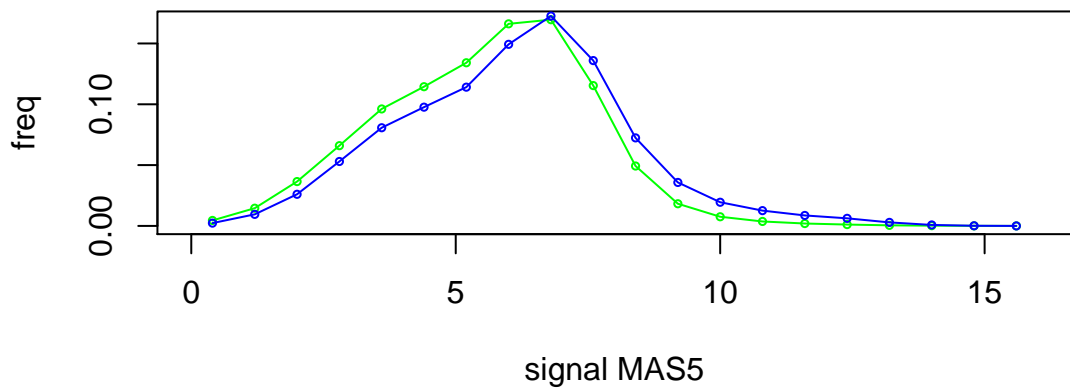

Supplement: Additional File 11 — Distribution of the expression signal for MT and non-MT probesets after processing with RMA and MAS5. The plots (normalized distributions of summarized expression values) indicate a slight increase in the high signal values for MT probesets (blue) against non-MT probesets (green). [file 1471-2105-7-276-S11.pdf]
